# Supplementary figures and images for: Mental health and addiction health service use by physicians compared to non-physicians before and during the COVID-19 pandemic: A population-based cohort study in Ontario, Canada
Source: PLoS Med. 2023 Apr 18;20(4):e1004187. doi: 10.1371/journal.pmed.1004187 (PMC10112788; doi:10.1371/journal.pmed.1004187)

**
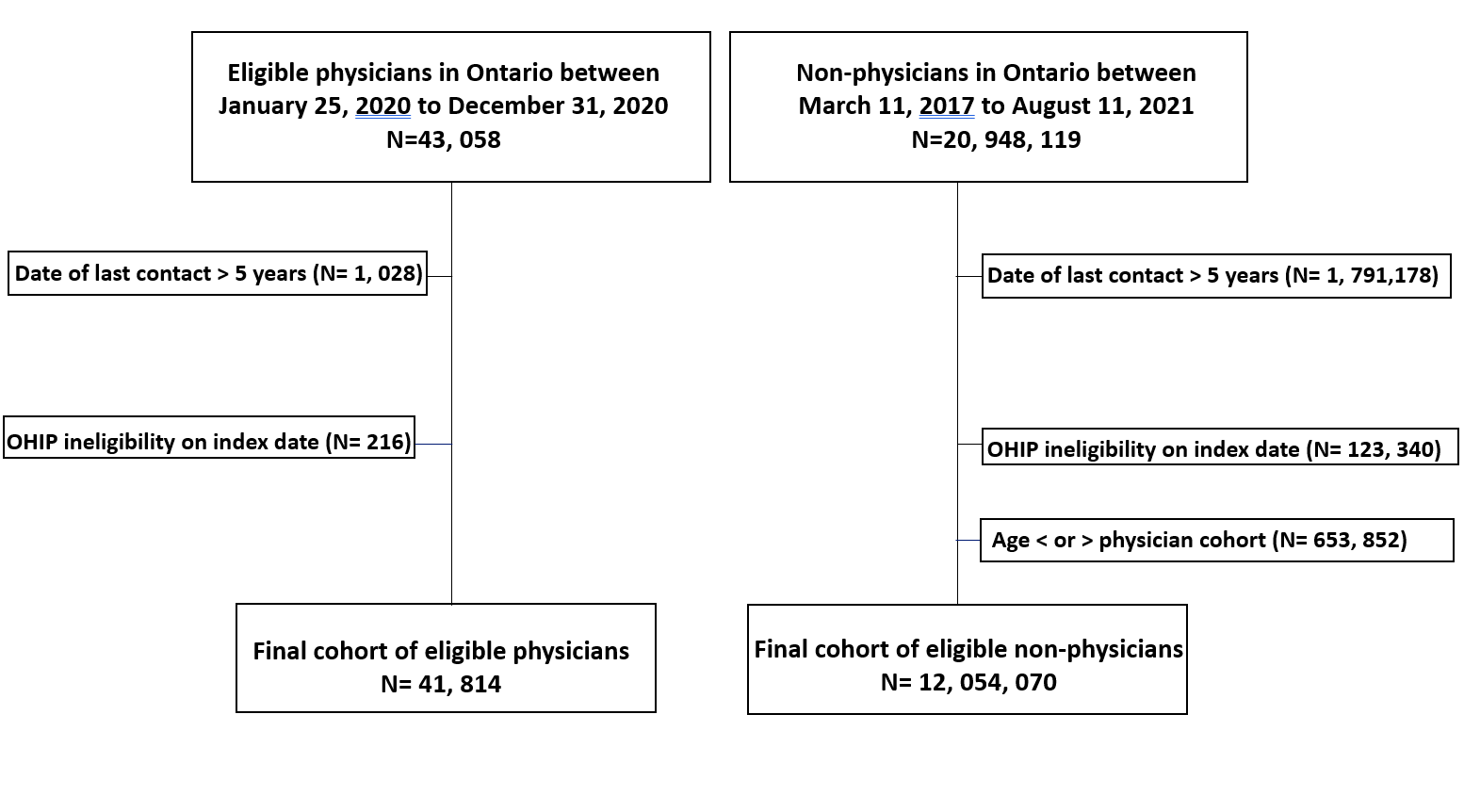
**

# **S1 Fig.** Study Cohort Build.

Supplement: S1 Fig — (DOCX) [file pmed.1004187.s002.docx]
