# Supplementary material for: Mental health and addiction health service use by physicians compared to non-physicians before and during the COVID-19 pandemic: A population-based cohort study in Ontario, Canada
Source: PLoS Med. 2023 Apr 18;20(4):e1004187. doi: 10.1371/journal.pmed.1004187 (PMC10112788; doi:10.1371/journal.pmed.1004187)
Supplement: S1 Dataset Creation Plan — (DOCX) [file pmed.1004187.s003.docx]

| Project InitiationThis Section must be Completed Prior to Project Dataset(s) Creation | | | | | |
| --- | --- | --- | --- | --- | --- |
| **Project Title:** | Physician mental health and wellness during COVID-19 | | | | |
| **Project TRIM number:** | 2021 0906 425 000 (originally 2021 0901 284 000) | | | | |
| **Research Program:** | KDT | | | | |
| **Site:** | ICES Western | | | | |
| **Project Objectives:** | *Insert Project Objectives as listed in the approved ICES Project PIA* | | | | |
|  | Report the incidence of outpatient mental health and substance use visits among physicians and non-physicians in Ontario before and during the COVI-19 pandemic | | | | |
| **ICES Project PIA Initial Approval Date:** | *The ICES Employee or agent who is responsible for creating the Project Dataset(s) is responsible for ensuring there is an approved ICES Project PIA and verifying the date of approval prior to creating the Project Dataset(s)* | | | | |
|  | 2020-Apr-04 | | | | |
| **Principal Investigator (PI):** | Peter Tanuseputro | | | | |
| **Check the applicable box if the PI is an ICES Student/Trainee** | ICES Student  ICES Fellow  ICES Post-Doctoral Trainee  Visiting Scholar | | | | |
| **Responsible ICES Scientist:** | *Name the Responsible ICES Scientist if the PI is not a Full Status ICES Scientist* | | | | |
|  | N/A | | | | |
| **ICES Project Team Member(s) Responsible for Project Dataset Creation and/or Statistical Analysis and date joined (list all):** | *All person(s) (ICES Analyst, Appointed Analyst, Analytic Epidemiologist, PI, and/or Student) responsible for creating the Project Dataset(s) and/or statistical analysis on the Research Analytics Environment (RAE) and the date they joined the project must be recorded* | | | | |
|  | Daniel Myran  Manish Sood  Amit Garg  Michael Pugliese  Mary Scott  Eric McArthur  Nivethika Jeyakumar | | | 2021-Feb-03 | |
| **Other Project Team Members and date joined (list all):** | *All other Research Project Team Members (e.g., Research Administrative Assistants, Research Assistants, Project Managers, Epidemiologists) and the date they joined the project must be recorded* | | | | |
|  |  | | | yyyy-mon-dd | |
| **Confirmation that DCP is consistent with Project Objectives:**  **NOTE: Project cannot start if this section is not complete.** | *The following individuals must confirm that the ICES Data provided for in this DCP is relevant (e.g., with respect to cohort, timeframe, and concepts) and required to achieve the Project Objectives stated in the ICES Project PIA prior to initial Project Dataset creation: 1) PI; 2) Responsible ICES Scientist if the PI is not a Full Status ICES Scientist, or a second ICES Scientist or the Scientific Program Lead if the PI is creating both the DCP and the Project Dataset[s]; 3) ICES Research Practice Staff creating the DCP; and 4) ICES Analytic Staff (ICES Employee or agent responsible for creating the Project Dataset[s]). This may be delegated either verbally or via e-mail.* | | | | |
|  | ***Principal Investigator*** | |  | | yyyy-mon-dd |
|  | ***Responsible ICES Scientist or Second ICES Scientist/Lead*** | |  | yyyy-mon-dd | |
|  | ***ICES Research Practice Staff Creating the DCP***  Nivethika Jeyakumar | |  | 2021-10-13 | |
|  | ***ICES Analytic Staff***  Eric McArthur | |  | 2021-10-19 | |
| **Designated ICES Research Practice Staff accountable for Project Documentation:** | *The person named (ICES staff) is accountable for ensuring that the approved ICES Project PIA, ICES Project PIA Amendments, and DCP are saved on the T Drive, ensuring ICES Project PIA Amendments are submitted as required, ensuring DCP Amendments are documented, and sharing the final DCP with the PI/Responsible ICES Scientist at project completion* | | | | |
|  |  | | | | |
| **DCP Creation Date and Author:** | *Date DCP was finalized prior to Project Dataset(s) creation* | *Name of person who created the DCP* | | | |
|  | ***Date*** | ***Name*** | | | |
|  | 2021-Sep-20 | Nivethika Jeyakumar | | | |

| ICES DataThis Section must be Completed Prior to Project Dataset(s) Creation | |
| --- | --- |
| *The ICES Employee or agent who is responsible for creating the Project Dataset(s) must ensure that this list includes only data listed in the ICES Project PIA.Changes to this list after initial ICES Project PIA approval require an ICES Project PIA Amendment.* | *Mandatory for all datasets that are available by individual year* |
| ***General Use Datasets – Health Services*** | ***Years (where applicable)*** |
| OHIP | 2015-2021 |
| ***General Use Datasets – Population*** |  |
| RPDB | 2017-2021 |
| ***General Use Datasets – Coding/Geography*** |  |
| LHIN | 2017-2021 |
| ***Other Datasets*** |  |
| OLIS | 2020-2021 |
| CPSO | 2020 |
|  |  |

| Project Amendments and Reconciliation | | | | |
| --- | --- | --- | --- | --- |
| **ICES Project PIA Amendment History (add additional rows as needed):** | *Privacy approval date* | *Person who submitted amendment* | *Note that any changes to the list of ICES Data or Project Objectives require an ICES Project PIA Amendment* |  |
|  | ***Date*** | ***Name*** | ***Amendment*** |  |
|  | yyyy-mon-dd |  |  |  |
| **DCP Amendment History:** |  | | | |
| **Date Programs/DCP reconciled** | *The person(s) creating the dataset and/or analyzing the data are responsible for ensuring that the final DCP reflects the final program(s) when the project is completed* | | | |
|  | yyyy-mon-dd | | | |

| Study Design and Project Time Frame Definitions | | |
| --- | --- | --- |
| **Study Design** | Cohort study  Matched cohort study  Case-control study  Cross-sectional study  Other (specify): Descriptive Study |  |
| **Project Timeline**  Look-back Window  Observation Window  (in which to look for outcomes)  **Index Event Date**  Accrual Window  Max Follow-up Date | |  |
| **Accrual Start/End Dates** | March 11, 2017 to August 11, 2021 |  |
| **Max Follow-up Date** | September 10, 2021 |  |
| **When does observation window terminate?** | - Death - Maximum follow-up period (September 10, 2021)   **Note**: Monthly cohorts will end before the start of the next interval |  |
| **Lookback Window(s)** | - 5 year lookback for comorbidities - 2 year health care utilization |  |

| Cohort Build | | |
| --- | --- | --- |
| *Note: Include a cohort build table in appendices.* | | |
| **Index Event / Inclusion Criteria and Index Date** | **Cohort 1:**  All active registered physicians from December 31, 2019, including new registrants up up until August 17, 2020 with valid IKNs.  **Cohort 2**  All non-physician adults in Ontario between March 11^th^ 2017 and August 11^th^ 2021.  **Index Date**: 11^th^ of every month (starting March 11^th^, 2017), we will be building monthly cohorts | |
| **Estimated Size of Cohort**  **(if known)** | N/A | |
| **Interval** | Monthly | |
| **Time Frame** | March 11, 2017 to September 10, 2021 | |
| **Exclusions (in order)** | **Step** | **Description** |
|  | 1 | Data cleaning exclusions for both cohorrs:   - Missing or invalid, age, or sex - Non-Ontario resident (individuals without RPDB variable   “prdcddablk” beginning with “35”) at index date   - Death on or before index date - Age < 18 years at index date - DOLC missing or > 5 years prior to index date - OHIP eligible on index date |
|  | ***Exclusions specific to non-physician cohort*** | |
|  | 2 Exclude non-physicians with an age < the youngest age in the physician cohort or >  oldest age in the physician cohort | |

| Concept Definitions (add additional rows as needed) | | |
| --- | --- | --- |
| *Note: Include concept definition details in appendices.* | |  |
| **Main Exposure or Risk Factor** | - COVID-19 Pandemic - Physician vs Non-Physician |  |
| **Primary Outcome Definition** | - Outpatient Mental Health and Substance Use Visits |  |
| **Secondary Outcome Definition(s)** | - Family Physician visits related to MHSU - Psychiatry visits related to MHSU |  |
| **Baseline Characteristics (by timeframe, e.g. at index, in the past year, etc.)** | Baselines will be provided at 2 time points to identify potential changes in the cohort.   - Beginning of the study (March 11, 2017) - Last monthly interval (August11, 2021)   See **Appendix B Table C2** for details |  |

| Analysis Plan and Dummy Tables **(Below is a guide – please MODIFY/EXPAND as appropriate)** |
| --- |
| Step 0: Cohort codes Review cohort code list   - Use %assign to obtain the cohort code list - Use %dinexplore to explore the set of DINs provided - PI to record date that they reviewed and approved of the cohort code list (quality assurance box below)   *****STOP FOR REVIEW*****  1. Cohort build **(Appendix B: Tables 1 cohort population). Details provided in Table C1.**   - Report the number of unique physicians and non-physicians records included during the total study period (March 11, 2017 to August 11, 2021) - **Manuscript S1 Fig.Study Cohort Build**   *****STOP FOR REVIEW*****  2. Baseline characteristics (**Appendix B:** **Table 2 Baselines**). Details provided in **Table C2.**   - Report baseline characteristics of individuals eligible for the monthly cohort for the following time period:   - Day before the start of the pandemic period (March 10, 2020)   - **Manuscript Table 1. Characteristics of physician and non-physicians on March 11, 2020.**   *****STOP FOR REVIEW*****   1. Preliminary look at monthly outcomes  - Report the number of individuals (physicians and non-physicians combined) (%) with at least one encounter for each outcome described in **Table C3.** Report the results for the pre-COVID time period and COVID time period separately. (**Appendix B: Table 3 Aggregate**) - Report monthly totals (N%) and rates per 1000 person-years for all outcomes described in Appendix B **Table C3.**  (**Appendix B: Tables 4-6**) - Create simple graphs plotting separate lines (physicians vs non-physicians) to visually explore whether there is an indication for a change between the pre-pandemic and pandemic period.   *****STOP FOR REVIEW*****   1. Age and Sex Standardized outpatient MHSU visit rates 2. Use indirect standardizationto report age and sex standardized monthly totals (N%) and rates per 1000 person-years for all outcomes described above.   Males: 18 to < 30, 30 to < 35, 35 to < 40, 40 to < 45, 45 to < 50, 50 to < 55, 55 to < 60, 60 to < 65, ≥65  Females: 18 to < 30, 30 to < 35, 35 to < 40, 40 to < 45, 45 to < 50, 50 to < 55, 55 to < 60, 60 to < 65, ≥65  **Note:** Age, sex standardize the physician rates based on the age, sex distribution in the general population.   - - **ManuscriptTable 2. Rates of Mental Health and Addiction Visits in Physician and non-physicians in the 36 months pre-pandemic (March 11, 2017- March 10, 2020) and during the first 18 months of the pandemic (March 11, 2020 – August 11, 2021).**  1. Create simple graphs with separate lines to visually explore any potential trends.    - Repeat step 3a ad b for for psyciatist and non-psychiatrist physicians **Manuscript S5 Table. Rates of outpatient health and addiction visits in psychiatrist physicians and non-psychiatrist physicians in the 36 months pre-pandemic (March 2017- February 2020) and during the first 18 months of the pandemic (March 2020 – August 2021).**   *****STOP FOR REVIEW*****   1. Subgroup Analyses for the primary outcome 2. Stratify monthly rates for both physicians and non-physicians by baseline MHSU visits in the 2 years prior to inerval date ( yes vs no)   *****STOP FOR REVIEW*****  **Additional Analytic Requests**   1. Repeat step 3a-c and 4a-b for acute care visits related to mental health defined as:   • Any OMHRS record (including missing, except for 290.x, 294.x in primary diagnosis). Excluded if primary diagnosis missing and provisional=17. (Source: OMHRS)  • A primary diagnosis of F06-F99, any diagnosis X60-X84, Y10-Y19, Y28 when the primary diagnosis is not F06-F99 (Source: DAD/NACRS)  **Note:** we might have small numbers in our monthly cohorts, so we might have to aggreagre to quarterly or semi-annual cohorts   - - **Manuscript Table 2. Rates of Mental Health and Addiction Visits in Physician and non-physicians in the 36 months pre-pandemic (March 11, 2017- March 10, 2020) and during the first 18 months of the pandemic (March 11, 2020 – August 11, 2021).**  1. Repeat steps 3a-c and 4a-b for virtual care visits vs non virtual care visits related to mental health   Note: Virtual care visit defined as having an outpatient MH visit (primary outcome) and an OHIP fee code equal to either “B100”, “B200”, “B099”, “B103”, “B203”, “B209”, “K080”, “K081”, “K082”, “K083”, “H409”, “H410” on the same servdate.  **Note:** we will have small numbers in our monthly cohort, so we might have to aggreagre to quarterly or semi-annual cohorts   - - **Manuscript Table 2. Rates of Mental Health and Addiction Visits in Physician and non-physicians in the 36 months pre-pandemic (March 11, 2017- March 10, 2020) and during the first 18 months of the pandemic (March 11, 2020 – August 11, 2021).**   *****STOP FOR REVIEW***** |
| Additional Analytic Requests (March 22, 2022) 9. Prior to pre-sampling of non-physicans for modelling analyses, exclude the following individuals from our cohort:   - Physicians and non-physicians who have missing rurality data - Non-physicians who have missing income quintile - For physicians who have a missing income quintile, impute the value 5 (highest income quintile) - After exclusions/imputations are applied, randomly select non-physicians to match the number of patients in our final physician cohort - Report baseline characteristics separately for all eligible non -physicians and the randomly selected non-physicans for the following time period: - Day before the start of the pandemic period (March 10, 2020)   - **Manuscript S3 Table. Characteristics of all non-physicians in Ontario on March 10, 2020 and random sample of non-physicians taken on March 10, 2020.**   *****STOP FOR REVIEW*****   1. Poisson Regression   Perform individual level poisson regression with generalized estimating equations (GEE) to account for correlation within each individual with repeated events to determine the association between physican status/COVD-19 and our primary outcome (outpatient MHSU visits). Report the rate ratio and 95% confidence interval  **Model 1:** Physician status (yes, no), COVID period (yes, no), physician status* COVID period, annual quarters (March 11th – May 11th; June 11- Aug 11th; Sept 11th – Nov 11th; Dec 11th- Feb 11th)  **Model 2:** Age, sex, physician status, covid period, physician status* COVID period, annual quarters  **Model 3:** Age, sex, income quintile, rurality, physician status, covid period, physician status* COVID period, annual quarters  **Model 4:** Age, sex, income quintile, rurality, prior MH (outpatient or acute), physician status, covid period, physician status* COVID period, annual quarters   - - **Manuscript Table 3. Poisson Regression Models comparing differences pre-COVID-19 and changes during COVID-19 pandemic in Mental Health and Addiction Visits between physicians and non-physicians.**   - **Manuscript S8 Table. Adjusted Poisson Regression Models comparing differences pre-COVID-19 and changes during COVID-19 pandemic in Mental Health and Addiction Visit Type between physicians and non-physicians.**   *****STOP FOR REVIEW*****  11a. Repeat step 10 for the following secondary outcomes:   - Family Physician visits related to MHSU - Psychiatry visits related to MHSU - Virtual outpatient MHSU visit - Non-virtual MHSU visit   - **Manuscript Table 3. Poisson Regression Models comparing differences pre-COVID-19 and changes during COVID-19 pandemic in Mental Health and Addiction Visits between physicians and non-physicians.**   - **Manuscript S8 Table. Adjusted Poisson Regression Models comparing differences pre-COVID-19 and changes during COVID-19 pandemic in Mental Health and Addiction Visit Type between physicians and non-physicians.**   b. Exclude psychiatrists from the physician cohort and re-run step 10 and 11a   - - **Manuscript Table 3. Poisson Regression Models comparing differences pre-COVID-19 and changes during COVID-19 pandemic in Mental Health and Addiction Visits between physicians and non-physicians.**   - **Manuscript S8 Table. Adjusted Poisson Regression Models comparing differences pre-COVID-19 and changes during COVID-19 pandemic in Mental Health and Addiction Visit Type between physicians and non-physicians.**   *****STOP FOR REVIEW*****   - Re-produce the following age and sex standardized figures: - Figure 1: Gen pop vs physicians- Outpatient Mental Health and Substance Use Visits   - **Manuscript Fig 1. Age and sex standardized monthly rates of outpatient mental health and addiction (MHA) visits overall, to psychiatrists, and to family physicians among physicians and non-physicians. Historic rates depict rates from March 2018 to August 2019.** - Figure 2: Gen pop vs physicians - Visit type (Family Physician visits related to MHSU vs Psychiatry visits related to MHSU) - Figure 3: Gen pop vs physicians- Virtual vs non-virtual MHSU visit   - **Manuscript Fig 2. Age and sex standardized monthly rates of virtual and in-person outpatient mental health and addiction visits among physicians and non-physicians.**   *****STOP FOR REVIEW*****   - Report the N (%) of Outpatient Mental Health and Addiction Related Codes for physicians and non-physicans during the pre-pandemic and pandemic period. Types of Outpatient MHA codes include: - Mental Health Codes - Anxiety, Somatoform, Dysthymia, Dissociative, Psychosomatic - Adjustment reaction, mood disorders, other mental health codes - Economic and social problem codes - Drug and alcohol use codes - Other psychiatrist codes   - **Manuscript S6 Table. Counts of Outpatient Mental Health and Addiction Related Codes by Physicians During the First 18 Months of the COVID-19 Pandemic Compared three years before the pandemic.** - Sensitivity Analysis - Exclude psychiatrists from the physician cohort and re-run step 10. - Exclude physicians and non-physicians who have a history of prior acute MH, and re-run **Model 4** in step 10 |

## Mnuscript Revison Requests (December 15, 2022)

1. Crude Poisson Models
2. Perform individual level poisson regression with generalized estimating equations (GEE) to account for correlation within each individual with repeated events to determine the association between physican status/COVD-19 and the secondary outcomes listed below. Report the rate ratio and 95% confidence interval.

- Psychiatry visits
- Family medicine visits
- Virtual care visits
- Acute care visits
  - **Manuscript S4 Table. Crude Poisson Regression Models comparing differences pre-COVID-19 and changes during COVID-19 pandemic in Mental Health and Addiction Visits between physicians and non-physicians.**

**Model 1:** Physician status (yes, no), COVID period (yes, no), physician status* COVID period, annual quarters (March 11th – May 11th; June 11- Aug 11th; Sept 11th – Nov 11th; Dec 11th- Feb 11th)

1. Exclude psychiatrists from the physician cohort and re-run step 14a
   - **Manuscript S4 Table. Crude Poisson Regression Models comparing differences pre-COVID-19 and changes during COVID-19 pandemic in Mental Health and Addiction Visits between physicians and non-physicians.**
2. Age spline for primary outcome

Perform individual level poisson regression with generalized estimating equations (GEE) to account for correlation within each individual with repeated events to determine the association between physican status/COVD-19 and our primary outcome (outpatient MHSU visits). Report the rate ratio and 95% confidence interval

**Model 5:** Age (spline), sex, income quintile, rurality, prior MH (outpatient or acute), physician status, covid period, physician status* COVID period, annual quarters

- - **Manuscript S7 Table. Poisson Regression Models comparing differences pre-COVID-19 and changes during COVID-19 pandemic in Mental Health and Addiction Visits between physicians and non-physicians.**

1. Negative Binomial Regression

Perform individual level negative binomial regression with generalized estimating equations (GEE) to account for correlation within each individual with repeated events to determine the association between physican status/COVD-19 and the primary outcome (outpatient MHSU visits). Report the rate ratio and 95% confidence interval.

**Model 1:** Physician status (yes, no), COVID period (yes, no), physician status* COVID period, annual quarters (March 11th – May 11th; June 11- Aug 11th; Sept 11th – Nov 11th; Dec 11th- Feb 11th)

| Quality Assurance Activities | | | |
| --- | --- | --- | --- |
| **RAE Directory of SAS Programs** |  | | |
| **RAE Directory of Final Dataset(s)** | *The* *final analytic dataset for each cohort includes all the data required to create the baseline tables and run all the models. It should include all covariates for all models such as patient risk factors, hospital characteristics, physician characteristics, exposure measures (continuous, categorical) and outcomes. It should include covariates that were considered but didn’t make the final cut. This would permit an analyst to easily re-run the models in the future.* | | |
|  |  | | |
| **RAE README file available:** Yes No | | | |
| **Date results of quality assurance tools for final dataset shared with project team (where applicable):** | | |  |
|  | | **%assign** | yyyy-mon-dd |
|  | | **%evolution** | yyyy-mon-dd |
|  | | **%dinexplore** | yyyy-mon-dd |
|  | | **%track / %exclude** | yyyy-mon-dd |
|  | | **%codebook** | yyyy-mon-dd |
| **Additional comments:** | |  | |

| Appendices (add appendices as needed) | |
| --- | --- |
| **Appendix A: Codes** | Refer to the “DCP Appendix A COVID-19 and MHSU Codes” Excel document   |
| **Appendix B: Output Tables** | Refer to the “DCP Appendix B COVID-19 and MHSU Output Tables” Excel document   |

**Appendix C: Concept Details**

**Table C1. Inclusion/Exclusion Criteria**

| **Inclusion/**  **Exclusion Criteria** | **Data Sources** | **Variables/**  **Code Types** | **Window** | **Notes**  **(including algorithm details)** |
| --- | --- | --- | --- | --- |
| ***Physician Cohort Inclusion Criteria*** | | | | |
| All physicians registered in CPSO with valid IKNs | CPSO | IKN |  |  |
| ***General Cohort Inclusion Criteria*** | | | | |
| All non-physician adults | RPDB |  | March 11, 2017 to August 11, 2021 | **Note:** Restrict to IKN not in CPSO dataset |
| ***Exclusion Criteria for both physician and non-physician cohorts*** | | | | |
| Invalid or missing, date of birth or sex | RPDB | SEX  BDATE | Ref date=Index date |  |
| non-Ontario resident | RPDB | PRCDDABLK | Ref date=Index date |  |
| Death on or before index date | RPDB | DTHDATE | DTHDATE≤ index date |  |
| Age < 18 on index date | RPDB | BDATE | Ref date=Index date |  |
| Date of Last Contact > 5 years | CIHI-DAD/SDS/NACRS  OHIP |  |  |  |
| OHIP eligible | OHIP |  | Ref date=Index date | %ohipelig macro |
| ***Exclusion Criteria specific to non-physician cohort*** | | | | |
| age < the youngest age in the physician cohort or > oldest age in the physician cohort |  |  |  |  |

**Table C2. Exposure and Baseline Characteristics**

| **Baseline Characteristic** | **Data Sources** | **Variables/**  **Code Types** | **Window** | **Reporting Detail** | **Notes**  **(including algorithm details)** |
| --- | --- | --- | --- | --- | --- |
| Age, years | RPDB | BDATE | Ref date=Index date | Mean (SD)  Median (IQR)  N (%): categories   1. 18 to <30 2. 30 to <35 3. 35 to < 40 4. 40 to < 45 5. 45 to < 50 6. 50 to < 55 7. 55 to < 60 8. 60 to < 65 9. ≥65 |  |
| Sex | RPDB |  | Ref date=Index date | N (%) female |  |
| Income quintile | RPDB_PSTLYEAR_LEVEL1 | INCQUINT | Ref date=Index date | N (%) in each quintile  1.  2.  3.  4.  5. | Categorize missing as income quintile 3  PSTLYEAR (using %getdemo) |
| Rural | RPDB | RURAL | Ref date = index date | N(%) Yes | categorize missing as urban  PSTLYEAR (using %getdemo) |
| LHIN | RPDB_PSTLYEAR_LEVEL1 | LHIN | Ref date = index date | N (%) in each LHIN  1.Erie St. Clair  2.South West  3.Waterloo Wellington  4.Hamilton Niagara Haldimand Brant  5.Central West  6.Mississauga Halton  7.Toronto Central  8.Central  9.Central East  10.South East  11.Champlain  12.North Simcoe Muskoka  13.North East  14.North West | PSTLYEAR (using %getdemo) |
| ***Physician Specific Characteristics*** | | | | | |
| Physician Specialty | CPSO | Specialty Type  Registration Class | Ref date = index date | N(%) in each category   1. Family Medicine 2. IMedicine 3. Emergency Medicine/Critical care 4. Anaesthesiology 5. Pediatrics 6. Surgery 7. Psychiatry 8. Other 9. Missing | See **Appendix A Tab BC_SPECIALTY** for details |
| Type of Registration Class | CPSO | Registration Class | Ref date = index date | N (%) in each category  1. Independent Practice  2. Postgraduate Education  3. Restricted  4. Academic Practice  5. Academic Visitor  6. Short Duration | See **Appendix A Tab BC_REG** for details |
| Canadian medical school graduate | CPSO | Medical School | Ref date = index date | N(%) in each category   1. Canadian 2. Non-Canadian 3. Missing | See **Appendix A Tab BC_CMG** for details |
| Years since Medical Graduation | CPSO | Graduation Year | Ref date = index date | Mean (SD)  Median (IQR) | **Note:** Index date 1- graduation year |
| Initial CPSO Registraion Date | CPSO | Initial CPSO Registraion Date | Ref date = index date | N(%) in each category   1. Before 1990 2. 1990 to < 2000 3. 2000 to < 2010   ≥ 2010 |  |
| LTC Practice Location | OHIP | PHYSNUM  LOCATION | Ref date = index date |  | **Note:** Restrict OHIP records using PHYSNUM and LOCATION= “L” |
| Front Line COVID-19 Care |  |  | During COVID-19 |  | For now binary yes vs no  If numbers permit  Divide into 0 patients seen  1-5 patients seen  6+ patient seen |
| ***Comorbidities*** | | | | | |
| John’s Hopkins ADGs |  |  | Ref date = index date | N(%) in each category   1. 0 2. 1-5 3. 6-9 4. ≥10 | Note: Lookback 2 years for measure |
| ***Health care utilization (2 year lookback from index date, not including index date)*** | | | | | |
| Outpatient Mental Health and Substance Use Visits | OHIP | FEECODE  DXCODE  SPEC  LOCATION | Ref date = index date |  | **Note:** Restrict to one visit per PHYSNUM per SERVDATE per IKN  Outpatient MHSU visits defined as   1. Psychiatrist visits defined as:   LOCATION= “O”, “L”, “H”, or “P” and SPEC= “19”  OR   1. MSHU related Family physician visits defined as:   LOCATION= “O”, “L”, “H”, or “P” and SPEC= “00” and codes found in **Appendix A Tab BC_MHSU** for details |

**Table C3. Outcomes**

| **Outcome Concept** | **Data Sources** | **Variables/**  **Code Types** | **Window** | **Reporting Detail** | **Notes**  **(including algorithm details)** |
| --- | --- | --- | --- | --- | --- |
| *Primary Outcome* | | | | | |
| Outpatient Mental Health and Substance Use Visits | OHIP | FEECODE  DXCODE  SPEC  LOCATION | Ref date=Index date to 10^th^ of next interval month | N (%) at least one encounter | **Note:** Restrict to one visit per PHYSNUM per SERVDATE per IKN. If multiple providers are seen on the same day, allow individuals to contribute one visit per provider (e., a patient who saw a primary care physician for a mental health-related reason and a psychiatrist on the same day would contribute two visits)  Outpatient MHSU visits defined as   1. Psychiatrist visits defined as:   LOCATION= “O”, “L”, “H”, or “P” and SPEC= “19”  OR   1. MSHU related Family physician visits defined as:   LOCATION= “O”, “L”, “H”, or “P” and SPEC= “00” and codes found in **Appendix A Tab BC_MHSU** for details |
| *Secondary Outcome(s)* | | | | | |
| Family Physician visits related to MHSU | OHIP | FEECODE  DXCODE  SPEC  LOCATION | Ref date=Index date to 10^th^ of next interval month | N (%) at least one encounter | **Note:** Restrict to one visit per PHYSNUM per SERVDATE per IKN. If multiple providers are seen on the same day, allow individuals to contribute one visit per provider (e., a patient who saw a primary care physician for a mental health-related reason and a psychiatrist on the same day would contribute two visits)  MSHU related Family physician visits defined as:  LOCATION= “O”, “L”, “H”, or “P” and SPEC= “00” and codes found in **Appendix A Tab OUT_MHSU** for details |
| Psychiatry visits | OHIP | FEECODE  DXCODE  SPEC  LOCATION | Ref date=Index date to 10^th^ of next interval month | N (%) at least one encounter | Psychiatrist visits defined as:  LOCATION= “O”, “L”, “H”, or “P” and SPEC= “19” |
