# Supplementary material for: Mental health and addiction health service use by physicians compared to non-physicians before and during the COVID-19 pandemic: A population-based cohort study in Ontario, Canada
Source: PLoS Med. 2023 Apr 18;20(4):e1004187. doi: 10.1371/journal.pmed.1004187 (PMC10112788; doi:10.1371/journal.pmed.1004187)
Supplement: S1 Text — (DOCX) [file pmed.1004187.s004.docx]

# **S1 Text.** Data Sources, Outcome/Covariate/Exposure, and Cohort Descriptions

**Data Sources:**

**Physician Linkage between CPSO and ICES:** Physicians were linked to health care visits using unique, encoded identifiers from the CPSO. Deterministic followed by probabilistic linkage (based on name, date of birth, and sex) was performed by a small, specialized group at ICES (formerly known as the Institute for Clinical and Evaluative Sciences). All identifying information was removed before data were sent to the study team. This is done to mitigate any privacy breaches. ICES is an independent, non-profit research institute that houses routinely collected health data from Ontario’s publicly funded health care system. ICES is a prescribed entity under section 45 of Ontario’s Personal Health Information Protection Act. Section 45 authorizes ICES to collect personal health information, without consent, for the purpose of health system evaluation and improvement. Projects conducted under section 45, by definition, do not require review by a specific institutional research ethics board. This project was conducted under section 45, and approved by ICES’s Privacy and Legal Office.

**Outcomes, Exposures and Covariates:** Physician demographic and speciality information was collected through the CPSO Registration Database and from the ICES Physicians Database. Data on physician and non-physician characteristics and health care use were obtained through the following linked databases at ICES: 1) the Ontario Registered Persons Database, which captured demographic information including age and sex; 2) the postal code conversion file which contains information on the rurality of an individual’s home address; 3) the OHIP Claims Database, which captured all outpatient claims for mental health visits, including virtual visits, in Ontario; and 4) the National Ambulatory Care Reporting System (NACRS), Ontario Mental Health Reporting System (OMHRS), and Discharge Abstract Database (DAD), which were used to capture acute care use as part of the definition for past history of mental health or substance use.

**Exclusions:** We excluded non-physicians with missing data on neighbourhood income-quintile or rurality. For physicians with missing data on neighbourhood income, we assigned them to the wealthiest income quintile.
