# Supplementary material for: Mental health and addiction health service use by physicians compared to non-physicians before and during the COVID-19 pandemic: A population-based cohort study in Ontario, Canada
Source: PLoS Med. 2023 Apr 18;20(4):e1004187. doi: 10.1371/journal.pmed.1004187 (PMC10112788; doi:10.1371/journal.pmed.1004187)
Supplement: S2 Text — (DOCX) [file pmed.1004187.s005.docx]

# **S2 Text-** Definition of MHSU Visits and type of visit

S1 Table lists the diagnostic and fee codes used to identify mental health and addiction (MHA) outpatient visits. These codes are from the Mental Health and Addictions Scorecard and Evaluation Framework indicator (MHASEF). We excluded the following codes from the MHASEF: tobacco use (OHIP diagnostic code 305), mental health codes related to pediatric presentations (OHIP Diagnostic Codes 313, 314, 315, and 319). To avoid double-counting, physicians or members of the general population with multiple MHA-related claims from one provider on the same day would only count as a single visit. Individuals who saw multiple providers on the same day were counted as one visit per provider (e.g.., a patient who saw a primary care physician and a psychiatrist on the same day would contribute two visits).

We identified outpatient MHA visits as virtual when the OHIP claim included either 1) a mental health-related diagnostic or fee code combined with a telemedicine flag or 2) a mental health-related diagnostic code and a corresponding virtual fee code.
