# Supplementary material for: Mental health and addiction health service use by physicians compared to non-physicians before and during the COVID-19 pandemic: A population-based cohort study in Ontario, Canada
Source: PLoS Med. 2023 Apr 18;20(4):e1004187. doi: 10.1371/journal.pmed.1004187 (PMC10112788; doi:10.1371/journal.pmed.1004187)
Supplement: S1 Table — (DOCX) [file pmed.1004187.s006.docx]

**Supplement**

**S1 Table.** List of OHIP Codes

| **Mental Health and Addiction Codes** | **Description of Code** |
| --- | --- |
| Diagnostic Codes | |
| 295 | Schizophrenia |
| 296 | Manic-depressive psychoses, involutional melancholia |
| 297 | Other paranoid states |
| 298 | Other psychoses |
| 300 | Anxiety neurosis, hysteria, neurasthenia, obsessive-compulsive neurosis, reactive depression |
| 301 | Personality disorders |
| 302 | Sexual deviations |
| 306 | Psychosomatic illness |
| 307 | Habit spasms, tics, stuttering, tension headaches, anorexia nervosa, sleep disorders, enuresis |
| 309 | Adjustment reaction |
| 311 | Depressive disorder |
| 897 | Economic problems |
| 898 | Marital difficulties |
| 899 | Parent-child problems |
| 900 | Problems with aged parents or in-laws |
| 901 | Family disruption / divorce |
| 902 | Education problems |
| 904 | Social maladjustment |
| 905 | Occupational problems |
| 906 | Legal problems |
| 909 | Other problems of social adjustment |
| 291 | Alcoholic psychosis, delirium tremens, Korsakov’s psychosis |
| 292 | Drug psychosis |
| 303 | Alcoholism |
| 304 | Drug dependence |
| Fee Codes | |
| A680 | Initial assessment – substance abuse |
| K680 | Substance abuse – extended assessment |
