# Supplementary material for: Mental health and addiction health service use by physicians compared to non-physicians before and during the COVID-19 pandemic: A population-based cohort study in Ontario, Canada
Source: PLoS Med. 2023 Apr 18;20(4):e1004187. doi: 10.1371/journal.pmed.1004187 (PMC10112788; doi:10.1371/journal.pmed.1004187)
Supplement: S2 Table — (DOCX) [file pmed.1004187.s007.docx]

# **S2 Table.** List of All Physician Specialities.

| **Study Specialty Groups *** | **CPSO Specialties** |
| --- | --- |
| Family Medicine | GP/FP, CCFP – FAMILY MEDICINE, CPSO – FAMILY MEDICINE |
| General Internal Medicine and Medicine Specialties** | INTERNAL MEDICINE, RCPSC – INTERNAL MEDICINE, NEUROLOGY, CARDIOLOGY, RCPSC – NEUROLOGY, RESPIROLOGY, GASTROENTEROLOGY, HEMATOLOGY, MEDICAL ONCOLOGY, ENDOCRINOLOGY, NEPHROLOGY, , RHEUMATOLOGY, GERIATRICS MEDICINE, INFECTIOUS DISEASES, RCPSC – RESPIROLOGY, RCPSC – MEDICAL ONCOLOGY, RCPSC – MEDICAL ONCOLOGY, RCPSC – CARDIOLOGY, RCPSC – NEPHROLOGY, RCPSC – ENDOCRINOLOGY AND METABOLISM, RCPSC – GASTROENTEROLOGY, RCPSC – HEMATOLOGY, RCPSC – INFECTIOUS DISEASES, CPSO – INTERNAL MEDICINE, CPSO – CARDIOLOGY, CPSO – MEDICAL ONCOLOGY, RCPSC – GERIATRIC MEDICINE, RCPSC – RHEUMATOLOGY, CPSO – HEMATOLOGY, CPSO – RESPIROLOGY, CPSO – GASTROENTEROLOGY, CPSO – INFECTIOUS DISEASES, CPSO – NEUROLOGY, CPSO – ENDOCRINOLOGY AND METABOLISM, RCPSC – CLINICAL PHARMACOLOGY AND TOXICOLOGY, CLINICAL PHARMACOLOGY |
| Surgery | GENERAL SURGERY, OBSTETRICS AND GYNECOLOGY, ORTHOPEDIC SURGERY, RCPSC – GENERAL SURGERY, OPHTHALMOLOGY, RCPSC – ORTHOPEDIC SURGERY, OTOLARYNGOLOGY, RCPSC – OBSTETRICS AND GYNECOLOGY, UROLOGY, PLASTIC SURGERY, RCPSC – OPHTHALMOLOGY, RCPSC – UROLOGY, NEUROSURGERY, CARDIAC SURGERY, RCPSC – NEUROSURGERY, RCPSC – OTOLARYNGOLOGY – HEAD AND NECK SURGERY, RCPSC – PLASTIC SURGERY, RCPSC – CARDIAC SURGERY, THORACIC SURGERY, VASCULAR SURGERY, RCPSC – VASCULAR SURGERY, PEDIATRIC SURGERY, CPSO – OBSTETRICS AND GYNECOLOGY, RCPSC – PEDIATRIC GENERAL SURGERY, RCPSC – THORACIC SURGERY, CPSO – CARDIAC SURGERY, CPSO – GENERAL SURGERY, CPSO – ORTHOPEDIC SURGERY, CPSO – OTOLARYNGOLOGY – HEAD AND NECK SURGERY, RCPSC – CARDIOVASCULAR AND THORACIC SURGERY, RCPSC – GENERAL SURGICAL ONCOLOGY, CPSO – OPHTHALMOLOGY, OTHER PGT – SURGICAL ONCOLOGY, RCPSC – PAEDIATRIC SURGERY, COLORECTAL SURGERY, SURGICAL ONCOLOGY GYNECOLOGIC ONCOLOGY, RCPSC – GYNECOLOGIC ONCOLOGY |
| Psychiatry | PSYCHIATRY, RCPSC – PSYCHIATRY, CPSO – PSYCHIATRY, RCPSC – CHILD AND ADOLESCENT PSYCHIATRY, RCPSC – FORENSIC PSYCHIATRY, RCPSC – FORENSIC PSYCHIATRY, RCPSC – GERIATRIC PSYCHIATRY, CPSO – FORENSIC PSYCHIATRY, CHILD & ADOLESCENT PSYCHIATRY, FORENSIC PSYCHIATRY, GERIATRIC PSYCHIATRY |
| Anesthesiology | ANESTHESIOLOGY, RCPSC – ANESTHESIOLOGY, CPSO – ANESTHESIOLOGY |
| Critical Care/Emergency Medicine | CRITICAL CARE, RCPSC – CRITICAL CARE MEDICINE, PEDIATRIC CRITICAL CARE, NO CERT – CRITICAL CARE MEDICINE, EMERGENCY MEDICINE, RCPSC – EMERGENCY MEDICINE, F.P./EMERGENCY MEDICINE, CCFP – FAMILY MEDICINE (EMERGENCY MEDICINE), PEDIATRIC EMERGENCY MEDICINE, RCPSC – PAEDIATRIC EMERGENCY MEDICINE, CPSO – EMERGENCY MEDICINE |
| Pediatrics | PEDIATRICS, RCPSC – PEDIATRICS, CPSO – PEDIATRICS, PEDIATRIC CLINICAL IMMUNOLOGY, PEDIATRICS CARDIOLOGY, RCPSC – PAEDIATRICS HAEMATOLOGY/ONCOLOGY, PEDIATRIC GASTROENTEROLOGY, PEDIATRIC NEUROLOGY, PEDIATRIC NEUROLOGY, PEDIATRIC HEMATOLOGY, PEDIATRIC INFECTIOUS DISEASES, PEDIATRIC NEPHROLOGY, PEDIATRIC RESPIROLOGY, ADOLESCENT MEDICINE, DEVELOPMENTAL PEDIATRICS, PEDIATRIC ENDOCRINOLOGY, PEDIATRIC RHEUMATOLOGY |
| Other | CPSO – PHYSICAL MEDICINE AND REHABILITATION, RCPSC – PHYSICAL MEDICINE AND REHABILITATION, PALLIATIVE MEDICINE, PHYSICAL MEDICINE AND REHAB,MEDICAL MICROBIOLOGY, RCPSC – PAIN MEDICINE, PAIN MEDICINE, RCPSC – MEDICAL GENETICS, RCPSC – OCCUPATIONAL MEDICINE, OCCUPATIONAL MEDICINE, MATERNAL FETAL MEDICINE, NO CERT – MATERNAL-FETAL MEDICINE, CPSO – NEONATAL-PERINATAL MEDICINE, NEONATAL/PERINATAL MEDICINE, RCPSC – MATERNAL FETAL MEDICINE, REPRODUCTIVE ENDOCRINOLOGY, RCPSC – GYNECOLOGIC REPRODUCTIVE ENDOCRINOLOGY & INFERTILITY, CPSO – DERMATOLOGY, CLINICAL IMMUNOLOGY, RCPSC – CLINICAL IMMUNOLOGY AND ALLERGY, RCPSC – MEDICAL MICROBIOLOGY, MEDICAL GENETICS, RCPSC – DERMATOLOGY, DERMATOLOGY, DIAGNOSTIC RADIOLOGY, RCPSC – DIAGNOSTIC RADIOLOGY, CPSO – DIAGNOSTIC RADIOLOGY, NO CERT – NEURORADIOLOGY, RCPSC – NEURORADIOLOGY, RCPSC – PAEDIATRIC RADIOLOGY, CPSO – PAEDIATRIC RADIOLOGY, FELLOW, FRCPC – FRCPC, ANATOMICAL PATHOLOGY, RADIATION ONCOLOGY, RCPSC – ANATOMICAL PATHOLOGY, RCPSC – RADIATION ONCOLOGY, FRCSC – FRCSC, GENERAL PATHOLOGY, LAB MEDICINE, NUCLEAR MEDICINE, HEMATOLOGICAL PATHOLOGY, RCPSC – HEMATOLOGICAL PATHOLOGY, RCPSC – GENERAL PATHOLOGY, RCPSC – NUCLEAR MEDICINE, RCPSC – NEUROPATHOLOGY, MEDICAL BIOCHEMISTRY, NEUROPATHOLOGY, CPSO – ANATOMICAL PATHOLOGY, RCPSC – FORENSIC PATHOLOGY, CPSO – RADIATION ONCOLOGY, CPSO – FORENSIC PATHOLOGY, CPSO – LABORATORY MEDICINE, FRCPSC – FRCPC(MSC), CPSO – GENERAL PATHOLOGY, CPSO – NEUROPATHOLOGY, RCPSC – MEDICAL BIOCHEMISTRY, FORENSIC PATHOLOGY, PEDIATRIC RADIOLOGY, COMMUNITY MED./PUBLIC HEALTH, RCPSC – COMMUNITY MEDICINE, RCPSC – PUBLIC HEALTH AND PREVENTIVE MEDICINE, CPSO – PUBLIC HEALTH AND PREVENTIVE MEDICINE |

* Note: The trainee/recent graduate group was defined as CPSO registrants with missing specialty information, where the year of graduation from medical school was between 2013 and 2018.

**Note: In Canada, General Internal Medicine (GIM)) is considered a subspeciality of internal medicine, and requires an extra year of training following three core years of internal medicine. General internists do not provide direct primary care and are only accessible by referral from another physician. For more information see: <https://link.springer.com/article/10.1007/s11606-016-3891-z>
