# Supplementary material for: Mental health and addiction health service use by physicians compared to non-physicians before and during the COVID-19 pandemic: A population-based cohort study in Ontario, Canada
Source: PLoS Med. 2023 Apr 18;20(4):e1004187. doi: 10.1371/journal.pmed.1004187 (PMC10112788; doi:10.1371/journal.pmed.1004187)
Supplement: S3 Table — (DOCX) [file pmed.1004187.s008.docx]

# **S3 Table.** Characteristics of all non-physicians in Ontario on March 10, 2020 and random sample of non-physicians taken on March 10, 2020.

| Variable | Non-Physicians  (n=10,972,726)^A^ | Non-Physician Random Sample  (n=39,380)^A^ |
| --- | --- | --- |
| Sex, No. (%) |  |  |
| Female | 5,650,764 (51.5%) | 20,192 (51.3%) |
| Male | 5,321,962 (48.5%) | 19,188 (48.7%) |
| Age in years, Mean (SD) | 50.5 (17.6) | 50.7 (17.7) |
| 18-34 years | 2,558,336 (23.3%) | 9,134 (23.2%) |
| 35-49 years | 2,829,019 (25.8%) | 10,085 (25.6%) |
| 50-64 years | 3,012,559 (27.5%) | 10,732 (27.3%) |
| 65+ years | 2,572,812 (23.5%) | 9,429 (23.9%) |
| Location or Home Address, No. (%) |  |  |
| Missing | 29,526 (0.3%) | 0 (0.0%) |
| Urban | 9,805,300 (89.4%) | 35,087 (89.1%) |
| Rural | 1,137,900 (10.4%) | 4,293 (10.9%) |
| Neighbourhood Income Quintile, No. (%) |  |  |
| Missing | 33,426 (0.3%) | 0 (0.0%) |
| 1 (Poorest) | 2,137,349 (19.5%) | 7,686 (19.5%) |
| 2 | 2,185,049 (19.9%) | 7,876 (20.0%) |
| 3 | 2,215,218 (20.2%) | 8,016 (20.4%) |
| 4 | 2,203,732 (20.1%) | 7,957 (20.2%) |
| 5 (Richest) | 2,197,952 (20.0%) | 7,845 (19.9%) |
| One or More Psychiatrist Visits in Past 2 Years | 518,034 (4.7%) | 1,846 (4.7%) |
| Psychiatry visits per year, mean (SD)^A^ | 7.0 (13.2) | 7.1 (11.7) |
| One or More Family Medicine Mental Health Visits in Past 2 years | 2,377,604 (21.7%) | 8,490 (21.6%) |
| Family Medicine visits per year, mean (SD)^A^ | 4.0 (8.8) | 4.0 (9.1) |
| One or More Acute Care Mental Health Visit in Past 2 years, No (%) | 64,811 (0.6%) | 240 (0.6%) |

^A^Population not equal to whole study population as some individuals had lost eligibility before March 10, 2020.

^B^Among individuals with 1 or more visits in past year.
