# Supplementary material for: Mental health and addiction health service use by physicians compared to non-physicians before and during the COVID-19 pandemic: A population-based cohort study in Ontario, Canada
Source: PLoS Med. 2023 Apr 18;20(4):e1004187. doi: 10.1371/journal.pmed.1004187 (PMC10112788; doi:10.1371/journal.pmed.1004187)
Supplement: S4 Table — (DOCX) [file pmed.1004187.s009.docx]

# **S4 Table.** Crude Poisson Regression Models comparing differences pre-COVID-19 and changes during COVID-19 pandemic in Mental Health and Addiction Visits between physicians and non-physicians.

| **MHA Visit Type** | **Population** | **Pre-COVID-19 Difference** | | **COVID-19 Change**  **(Reference = pre-COVID-19** | | |  |
| --- | --- | --- | --- | --- | --- | --- | --- |
|  |  | **Crude Incidence Rate Ratio** | **95% CI** | **Crude Incidence Rate Ratio** | | **95% CI** | |
| Overall | Physician | 1.12 | (1.04, 1.21) | 1.33 | (1.27, 1.40) | |  |
|  | Physician excluding psychiatry | 1.03 | (0.98, 1.13) | 1.38 | (1.32, 1.45) | |  |
|  | Non-Physician | Reference | [1] | 1.10 | (1.08, 1.13) | |  |
| Psychiatry | Physician | 3.63 | (3.35, 3.92) | 1.22 | (1.16, 1.29) | |  |
|  | Physician excluding psychiatry | 2.61 | (2.41, 2.84) | 1.33 | (1.15, 1.32) | |  |
|  | Non-Physician | Reference | [1] | 1.08 | (1.04, 1.12) | |  |
| Family Medicine | Physician | 0.65 | (0.61, 0.69) | 1.28 | (1.1.21, 1.36) | |  |
|  | Physician excluding psychiatry | 1.11 | (1.09, 1.13) | 1.33 | (1.26, 1.40) | |  |
|  | Non-Physician | Reference | [1] | 1.11 | (1.09, 1.13) | |  |
| Virtual Care | Physician | 0.36 | (0.26, 0.50) | 62.66 | (45.45, 86.39) | |  |
|  | Physician excluding psychiatry | - | - | - | - | |  |
|  | Non-Physician | Reference | [1] | 11.81 | (11.25, 12.40) | |  |
| Acute Care | Physician | 0.17 | (0.14, 0.20) | 1.01 | (0.80, 1.28) | |  |
|  | Physician excluding psychiatry | - | - | - | - | |  |
|  | Non-Physician | Reference | [1] | 0.96 | (0.88, 1.05) | |  |
