# Supplementary material for: Mental health and addiction health service use by physicians compared to non-physicians before and during the COVID-19 pandemic: A population-based cohort study in Ontario, Canada
Source: PLoS Med. 2023 Apr 18;20(4):e1004187. doi: 10.1371/journal.pmed.1004187 (PMC10112788; doi:10.1371/journal.pmed.1004187)
Supplement: S5 Table — (DOCX) [file pmed.1004187.s010.docx]

# **S5 Table.** Rates of outpatient health and addiction visits in psychiatrist physicians and non-psychiatrist physicians in the 36 months pre-pandemic (March 2017- February 2020) and during the first 18 months of the pandemic (March 2020 – August 2021).

|  |  | **Pre-COVID19 Pandemic** | | **During COVID19 Pandemic** | | **Crude Relative Change in Rate of Visits**  **(%)** |
| --- | --- | --- | --- | --- | --- | --- |
|  |  | No. Visits | Visits Per 1,000 Person Years | No. Visits | Visits Per 1,000 Person Years |  |
| Overall | Psychiatrist Physicians | 24,642 | 4,219.1 | 13,552 | 4455.5 | 27.4 |
|  | Non-Psychiatrist Physicians | 71,148 | 697.7 | 53,537 | 919.2 | 5.5 |
| Psychiatry | Psychiatrist Physicians | 20,937 | 3584.7 | 11,748 | 3862.4 | 7.5 |
|  | Non-Psychiatrist Physicians | 44,630 | 437.6 | 33,605 | 577.0 | 31.8 |
| Family Medicine | Psychiatrist Physicians | 3,705 | 634.5 | 1804 | 593.1 | -6.5 |
|  | Non-Psychiatrist Physicians | 26,518 | 260.0 | 19,932 | 342.2 | 31.6 |
