# Supplementary material for: Mental health and addiction health service use by physicians compared to non-physicians before and during the COVID-19 pandemic: A population-based cohort study in Ontario, Canada
Source: PLoS Med. 2023 Apr 18;20(4):e1004187. doi: 10.1371/journal.pmed.1004187 (PMC10112788; doi:10.1371/journal.pmed.1004187)
Supplement: S6 Table — (DOCX) [file pmed.1004187.s011.docx]

# **S6 Table.** Counts of Outpatient Mental Health and Addiction Related Codes by Physicians During the First 18 Months of the COVID-19 Pandemic Compared three years before the pandemic.

|  | **Physicians** | | **Non-Physicians** | |
| --- | --- | --- | --- | --- |
| **Time Period** | **Pre-COVID-19** | **During COVID-19** | **Pre-COVID-19** | **During COVID-19** |
|  | **Count of codes (% of all codes)** | | **Count of codes (% of all codes)** | |
| Total Codes (OHIP or Billing Code) | 95,790 | 67,137 | 19,816,019 | 11,285,102 |
| Mental Health Codes | 86,870 (90.6%) | 60,835 (90.6%) | 14,848,900 (74.9%) | 8640871 (76.6%) |
| Anxiety, Somatoform, Dysthymia, Dissociative, Psychosomatic (300) | 65,849 (68.7%) | 46,058 (68.6%) | 9,318,735 (47.0%) | 5,536,885 (49.1%) |
| Adjustment reaction (309) | 3,252 (3.4%) | 2,723 (4.1%) | 436,575 (2.2%) | 262,603 (2.3%) |
| Mood disorders (296, 311) | 13,921 (14.5%) | 9,480 (14.1%) | 3,111,799 (15.7%) | 1,723,095 (15.3%) |
| Other Mental Health Codes (295, 297, 298, 301, 302, 306, 307) | 3,848 (4.0%) | 2,574 (3.8%) | 1,981,791 (10.0%) | 1,118,288 (9.9%) |
| Economic and Social Problem Codes (897, 902, 904 - 906, 909) | 2,181 (2.3%) | 1,118 (1.7%) | 401,360 (2.0%) | 199,563 (1.8%) |
| Drug and Alcohol Use Codes (291, 292, 303, 304, A680, K680) | 6,036 (6.3%) | 3,730 (5.6%) | 4,351,897 (22.0%) | 2,319,465 (20.6%) |
| Other psychiatrist codes | 703 (0.7%) | 1,454 (2.2%) | 213,862 (1.1%) | 125,203 (1.1%) |
