# Supplementary material for: Mental health and addiction health service use by physicians compared to non-physicians before and during the COVID-19 pandemic: A population-based cohort study in Ontario, Canada
Source: PLoS Med. 2023 Apr 18;20(4):e1004187. doi: 10.1371/journal.pmed.1004187 (PMC10112788; doi:10.1371/journal.pmed.1004187)
Supplement: S7 Table — (DOCX) [file pmed.1004187.s012.docx]

# **S7 Table.** Poisson Regression Models comparing differences pre-COVID-19 and changes during COVID-19 pandemic in Mental Health and Addiction Visits between physicians and non-physicians.

| **Age specifical in Model** | **Population** | **Pre-COVID-19 Difference** | | **COVID-19 Change**  **(Reference = pre-COVID-19** | | |  |
| --- | --- | --- | --- | --- | --- | --- | --- |
|  |  | **Adjusted Incidence Rate Ratio^A^** | **95% CI** | **Adjusted Incidence Rate Ratio^A^** | | **95% CI** | |
| Age (years) Continuous | Physician | 1.00 | (0.91, 1.10) | 1.39 | (1.28, 1.51) | |  |
|  | Non-Physician | Reference | [1] | 1.12 | (1.09, 1.14) | |  |
| Age (years) Restricted Cubic Splines | Physician | 1.03 | (0.94, 1.13) | 1.37 | (1.30, 1.45) | |  |
|  | Non-Physician | Reference | [1] | 1.12 | (1.09, 1.14) | |  |

^A^Models adjusted for annual quarter, age (continuous or cubic spline), rurality, income-quintile, and sex (male vs female).
