# Supplementary material for: Mental health and addiction health service use by physicians compared to non-physicians before and during the COVID-19 pandemic: A population-based cohort study in Ontario, Canada
Source: PLoS Med. 2023 Apr 18;20(4):e1004187. doi: 10.1371/journal.pmed.1004187 (PMC10112788; doi:10.1371/journal.pmed.1004187)
Supplement: S8 Table — (DOCX) [file pmed.1004187.s013.docx]

# **S8 Table.** Adjusted Poisson Regression Models comparing differences pre-COVID-19 and changes during COVID-19 pandemic in Mental Health and Addiction Visit Type between physicians and non-physicians.

| **MHA Visit Type** | **Population** | **Pre-COVID-19 Difference** | | **COVID-19 Change** | |
| --- | --- | --- | --- | --- | --- |
|  |  |  |  | **(Reference = pre-COVID-19** | |
|  |  | **Beta coefficient** | **Standard Error** | **Beta coefficient** | **Standard Error** |
| Overall | Physician | 0.003 | 0.047 | 0.327 | 0.028 |
|  | Physician excluding psychiatry | -0.006 | 0.035 | 0.365 | 0.026 |
|  | Non-Physician | Reference | [1] | 0.11 | 0.012 |
| Psychiatry | Physician | 1.363 | 0.049 | 0.197 | 0.026 |
|  | Physician excluding psychiatry | 1.018 | 0.051 | 0.279 | 0.031 |
|  | Non-Physician | Reference | [1] | 0.078 | 0.019 |
| Family Medicine | Physician | -0.480 | 0.036 | 0.288 | 0.031 |
|  | Physician excluding psychiatry | -0.55 | 0.034 | 0.322 | 0.03 |
|  | Non-Physician | Reference | [1] | 0.108 | 0.011 |
| Virtual Care | Physician | -1.244 | 0.214 | 4.344 | 0.214 |
|  | Physician excluding psychiatry | - | - | - | - |
|  | Non-Physician | Reference | [1] | 2.459 | 0.025 |
| Acute Care | Physician | -1.674 | 0.093 | 0.025 | 0.122 |
|  | Physician excluding psychiatry | - | - | - | - |
|  | Non-Physician | Reference | [1] | -0.105 | 0.046 |

Models adjusted for annual quarter, age (continuous), rurality, income-quintile, and sex (male vs female).
