# Supplementary material for: Mental health and addiction health service use by physicians compared to non-physicians before and during the COVID-19 pandemic: A population-based cohort study in Ontario, Canada
Source: PLoS Med. 2023 Apr 18;20(4):e1004187. doi: 10.1371/journal.pmed.1004187 (PMC10112788; doi:10.1371/journal.pmed.1004187)
Supplement: Attachment — Submitted filename: Reviewer Response January 3, 2023.docx [file pmed.1004187.s014.docx]

January 3, 2022

RE: PMEDICINE-D-22-02286R1: "Outpatient mental health and addiction visits by physicians compared to non-physicians before and during the COVID-19 pandemic.”

Dear Callam Davidson and the PLOS Medicine Team.

Thank you for taking the time to review our manuscript *“*Outpatient mental health and addiction visits by physicians compared to non-physicians before and during the COVID-19 pandemic*”* and your interest in our work. Below please find our detailed response to the four peer reviewers.

Thank you for once again considering our work and we look forward to hearing back from you.

Daniel Myran, on behalf of the author team

Requests from the editors dated Dec 27, 2022

January 3, 2022

1. Please upload a file showing your changes either highlighted or using a track changes function in your word processor. This document should be uploaded as the item type 'Revised Article with Changes Highlighted.'

We have uploaded a copy with tracked changes. You can view these track changes by going to ‘Review’ and selecting ‘All mark up’

2. Please amend your detailed Financial Disclosure statement on the online submission form. This is published with the article. It must therefore be completed in full sentences and contain the exact wording you wish to be published.

In the manuscript we separated the Financial Disclosure from the Acknowledgement section as recommended. For the Financial Disclosure we included information on the grant (including titles, numbers (when applicable), organizations, and authors awarded). We included a statement that the funders had no roll in the study. We ensured that the Financial Disclosure in the manuscript matches that in the online submission.

3. We have amended your Competing Interest statement to comply with journal style. We kindly ask that you double check the statement and let us know if anything is incorrect.

Reviewed and accepted.

4. Please note that the contact for accessing your data cannot be an author on the manuscript. Please therefore replace the current contact information with a third party contact, such as an institutional data access committee.

We have changed the statement to contact the institution ICES.

5. We have noticed that you have uploaded Supporting Information files, but you have not included a list of legends. Please add a full list of legends for your Supporting Information files after the references list.

A list of Supporting Information Files is included in the Manuscript after the list of references.

Requests from the editors dated December 12, 2022:

1. Please revise your title according to PLOS Medicine's style: please place the study design ("A population-based cohort study") in the subtitle (ie, after a colon). To ensure clarity, we suggest "Outpatient mental health and addiction service use by physicians compared to non-physicians before and during the COVID-19 pandemic: a population-based cohort study", or similar.

**Author response:** This has been completed

2. Please include continuous line numbering throughout the manuscript to facilitate further review.

**Author response:** This has been completed

3. Abstract Methods and Findings: Please update the term ‘COVID-19 pandemic period’ to ‘the first 18 months of the COVID-19 pandemic’ or similar.

**Author response:** This has been completed

4. The Data Availability Statement (DAS) requires revision.
* The URL leads to an error page, please check and update as required.
* You state that ‘The full data set creation plan and underlying analytic code are available from the authors on request’. Please note that a study author cannot be the contact person for the data. See our website for suggested alternatives: <https://journals.plos.org/plosmedicine/s/data-availability>

We’ve updated the link, so it should now work.

Please note that all the study data is held at ICES. ICES is an independent, non-profit research institute whose legal status under Ontario’s health information privacy law allows it to collect and analyze health care and demographic data, without consent, for health system evaluation and improvement. While data sharing agreements prohibit ICES from making the data set publicly available, access may be granted by ICES to those who meet pre-specified criteria for confidential access, available at <https://www.ices.on.ca/DAS>. (ICES will review and approve data access requests).

The comment of “The full data set creation plan and underlying analytic code are available from the authors on request” pertains to the analysis plan and code used to conduct the study. As the “Data Availability Statement” pertains to the data and not the analyses plan, we have removed this wording to prevent confusion.

**Author response:** This has been completed

5. The funding information can be removed from the cover page – this information is captured via the submission form and will be published as metadata in the event of acceptance.

**Author response:** This has been completed

6. Please place citations within square brackets and preceding punctuation throughout your manuscript.

**Author response:** This has been completed

7. When citing your Supporting Information (e.g., Methods Supplement), please use the naming conventions outlined here: <https://journals.plos.org/plosmedicine/s/supporting-information>

**Author response:** This has been completed

8. Please define the abbreviation MHA in the Figure 1 legend.

**Author response:** We have placed this abbreviation definition in the figure caption please let us know if you would like it somewhere else.

9. Please provide the crude IRRs as well as the adjusted IRRs in Table 3 (or alternatively please report the crude analyses in the Supporting Information and cite these in the Results).

**Author response:** The crude IRRs have been added to the supplement, please see supplement eResults Table 2 and have been references in the text.

10. Please ensure that the study is reported according to the STROBE guideline, and include the completed STROBE checklist as Supporting Information. Please add the following statement, or similar, to the Methods: "This study is reported as per the Strengthening the Reporting of Observational Studies in Epidemiology (STROBE) guideline (S1 Checklist)."

The STROBE guideline can be found here: <http://www.equator-network.org/reporting-guidelines/strobe/>

When completing the checklist, please use section and paragraph numbers, rather than page numbers.

**Author response:** This has been completed

11. Did your study have a prospective protocol or analysis plan? Please state this (either way) early in the Methods section.

a) If a prospective analysis plan (from your funding proposal, IRB or other ethics committee submission, study protocol, or other planning document written before analyzing the data) was used in designing the study, please include the relevant prospectively written document with your revised manuscript as a Supporting Information file to be published alongside your study, and cite it in the Methods section. A legend for this file should be included at the end of your manuscript.

**Author response:** we have included the study protocol and data set creation plan that was written before analyzing the data. This has been uploaded as a supporting file and referenced in the methods.

b) If no such document exists, please make sure that the Methods section transparently describes when analyses were planned, and when/why any data-driven changes to analyses took place.

c) In either case, changes in the analysis-- including those made in response to peer review comments-- should be identified as such in the Methods section of the paper, with rationale.

**Author response:** At this point we have conducted a series of sensitivity analyses in response to Reviewer 1. Before including these changes in the methods section (e.g. analysis with age as a spline) we will wait for your comments on your preference for inclusion.

**Comments from the reviewers:**

**Reviewer #1:** Thanks for the opportunity to review your manuscript. My role is as a statistical reviewer, so my review concentrates on the study design, data, and analysis that are presented. I have put general questions first, followed by queries relevant to a specific section of the manuscript (with a page/paragraph reference).

This study is an observational cohort study that compares changes in utilisation of healthcare between physicians and general population from 2017 to 2021, specifically family medicine and outpatient mental health and addiction visits. The study uses routinely collected health data, linked to physician/surgeon registration data. The study uses a difference-in-difference approach, estimating the difference between physicians and non-physicians in the difference between pre-COVID and COVID time periods (done by including an interaction between a COVID time period indicator and physician indicator variable).

One of the strengths of the study is the size and coverage - the integrated data available from this province is impressive and I think is a demonstration of the value of building data infrastructure as this study would have been impossible to do prospectively.

1. It wasn't directly specified in the methods, but it looks as though age-groups included were relatively large (e.g. 35-49, 50-64). In terms of the effect of reducing confounding, this assumes that a 35 year old has the same levels of healthcare utilisation as a 49 year old. I would like to see a sensitivity analysis that includes age as a continuous variable in spline form to see if this reduces the differences between physicians and non-physicians as I suspect the age structure between the two groups within the 35-49 and the 50-64 age-groups could be different.

**Author response:** Thanks for the suggestion. Our analyses actually included age (in year) as a continuous variable. We have specified this in our methods more clearly. We have completed the sensitivity analysis for our primary outcome and present the results below. The results suggest no meaningful change in effect estimate or confidence intervals whether adjusting for age continuously per year or with restricted cubic splines. At the discretion of the editors, we are happy to change our adjustment for age to use restricted cubic splines in all models if requested. We are also happy to present this sensitivity analysis in supplement.

| **Age specifical in Model** | **Population** | **Pre-COVID-19 Difference** | | **COVID-19 Change**  **(Reference = pre-COVID-19** | | |  |
| --- | --- | --- | --- | --- | --- | --- | --- |
|  |  | **Incidence Rate Ratio^A^** | **95% CI** | **Incidence Rate Ratio^A^** | | **95% CI** | |
| Age (years) Continuous | Physician | 1.00 | (0.91, 1.10) | 1.39 | (1.28, 1.51) | |  |
|  | Non-Physician | Reference | [1] | 1.12 | (1.09, 1.14) | |  |
| Age (years) Restricted Cubic Splines | Physician | 1.03 | (0.94, 1.13) | 1.37 | (1.30, 1.45) | |  |
|  | Non-Physician | Reference | [1] | 1.12 | (1.09, 1.14) | |  |

^A^Models adjusted for annual quarter, age (continuous or cubic spline), rurality, income-quintile, and sex

2. How many (%) of the cohort (non-phys/phys) had missing information on neighbourhood income quintile? Were those missing this information different to those with complete data?

**Author response:** The number of individuals with missing neighborhood income quintile was low in both cohorts (0.5% physicians and 0.3%) non-physicians. We have added this to the manuscript. All physicians with missing income quintile data were assumed to reside in the highest income neighborhood given their socioeconomic status.

*“Data for the above covariates was complete except for income quintile which was missing for 0.5% of physicians and 0.3% of non-physicians. Physicians were missing data for income were assigned to the richest income quintile neighbourhood and non-physicians with missing data for income were excluded from regression models.”*

3. P8, Paragraph 2. Is the form of the covariates used in the main analyses the same as in table 1?

**Author response:** The covariates used in the main analysis were age (continuous in years), quarter, rurality, sex, and income quintile. This has now been specified more clearly in the Table 3 footnote and in our methods.

*“^A^Models adjusted for quarter, age (continuous), rurality, income-quintile, and sex”*

4. P8, Paragraph 3. What age-groups were used for the age standardisation?

**Author response:** We were able to standardize the physicians based on single-year age groups (i.e. a 40-year old physician was standardized based on 40-year old non-physicians).

5. P9, Paragraph 1. What type of covariance structure (e.g. exchangeable, AR(1)) was used in the GEEs?

**Author response:** We used an exchangeable covariance structure, this has been added to the methods.

*“Using the individual’s monthly interval as the unit of analysis, we conducted Poisson models using generalized estimating equations with an exchangeable covariance structure.”*

6. Was the suitability of the Poisson distribution assessed? E.g. any evidence of excess 0s, or overdispersion?

**Author response:** Potential goodness of fit measures for Poisson regression with count data such as zero-inflation and overdispersion were not assessed as the intent of this modelling was to produce valid likelihood-based estimates of the incidence rate ratio that would align with our crude rates observed. Zero-inflated and negative binomial models would not provide the same interpretation. As Berry has shown, “for likelihood purposes it is valid to treat X [outcome variable] as a Poisson variable…, even though X is not distributed as a Poisson variable.”

Berry, G. (1983). The analysis of mortality by the subject-years method. *Biometrics*, 173-184.

7. Was autocorrelation checked for?

**Author response:** Considering that individuals were accrued over a fairly short study period of 4 years and they were included at the start of each eligible index month (i.e. inclusion was not dependent on a healthcare encounter), we felt that assuming an exchangeable covariance structure was reasonable and did not test for autocorrelation or explore other structures such as AR(1). Further, prior simulation studies have shown that GEE models are generally robust to specification of the covariance structure.

Overall, J. E., & Tonidandel, S. (2004). Robustness of generalized estimating equation (GEE) tests of significance against misspecification of the error structure model. *Biometrical Journal: Journal of Mathematical Methods in Biosciences*, *46*(2), 203-213.

8. Why was a random sample of non-physicians used instead of just including all available data?

**Author response:** We used a random sample of non-physicians as the computing requirements of conducing a GEE analysis using more than 13 million individuals exceeds the capacity of .non-specialized computing environments. Using a random sample is general practice at ICES in these circumstances and in the supplement we show that the random sample of the general population does not differ from the entire general population on key covariates.

9. What criteria were you using to infer that there is a difference between time-periods or exposure groups? 95% CI

**Author response:** We used 95% CI with effect estimates not crossing the null. We have added this to the methods.

“We interpreted confidence intervals which did not cross 1 as a difference between time periods or exposure groups.”

At the editors discretion we are happy to calculate and present p values for the interaction terms (e.g. difference in changes in rates of outpatient MHA visits during COVID-19 between physicians and non-physicians).

10. P11, Table 1. I would add a 'No. (%)' to the age part of the table where this is presented by age-group.

**Author response:** This has been completed

11. P12 Paragraph 1. Can the regression results (e.g. beta coefficients) of these regression models used to estimate IRR be included as an appendix?

**Author response:** The beta coefficients were exponentiated to produce the IRRs in Table 3. At the editors discretion we are happy to include these an appendix.

12. P16. Paragraph 1. The change is virtual visits is enormous - were there changes in policy at the time to allow more patients to use these, or to use these instead of in-person visits where possible?

**Author response:** Yes, during our study time frame the were enormous changes to the delivery of virtual care. On March 19, 2020 the Ontario Ministry of Health issued a directive to health care providers to stop all nonessential or elective medical services including outpatient office visits. Shortly after the ministry of health authorized the use of temporary billing codes to facilitate virtual care by renumerating much broader use of telephone and virtual based care (e.g. pre-pandemic virtual care was only renumerated under specific in limited circumstances) which remained in effect for the study duration. We have added this context to the introduction

*“In addition, we examined differences in visit type (psychiatry, family medicine), in-person vs virtual visits, and acute care visits, as the COVID-19 pandemic led to policy changes that increased access to outpatient virtual care services by renumerating much broader use of telephone and virtual based care.”*

13. eTable 1. What is the category of the top row of each variable? Is this the missing data for that variable?

**Author response:** Yes, these numbers were the missing values for rural address and neighborhood income-quintile. We have revised this to indicate these are the missing values.

Reviewer #2: Mental health help seeking among physicians, particularly any changes over the pandemic, is an important topic that is often discussed without research to back it and this paper is the first to do so. The use of a population-based cohort and confirming physician status by registration, helps to limit any lack of disclosure of job title by physician's themselves for fear of stigma and creates a large cohort.

A few suggestions to help improve upon an already important paper:
- Introduction:

1. The paragraph on p.5 reads like a limitations paragraph instead of introductory paragraph and could be reworded to emphasize what you did, in the context of the limitations of prior work, instead of starting with the limitations.

**Author response:** Thank you for the suggestion. We have reworked this paragraph so that the limitations were shortened and moved to the prior paragraph to help highlight gaps in the literature. The final paragraph in the introduction now focuses solely on what we did.

2. Is there anything unique about the pandemic in Ontario that might have influenced the population as a whole, but physicians specifically, that readers need to know? Anything that might make the population any different from physicians anywhere else?

**Author response:** This is an important comment for generalizability. Like most peer high-income countries, the pandemic in Ontario resulted in substantial strain on the health system and health care workers. Our study time frame included three pandemic waves, the roll out of COVID-19 vaccines for health care workers and later the general population. We have added this context to the methods section to help an international audience contextualize the period in Ontario.

*The first 18 months of the COVID-19 pandemic in Ontario were broadly characterized by three periods of high levels of infection and consequent burden on the health system (March 2020 to May 2020, mid-September 2020 to February 2021, and March 2021 to May 2021) with two periods of relatively lower levels of infections (June 2020 to September 2020, and June 2021 to August 2021). Vaccines in Ontario became available to health care workers in the highest risk settings and residents of long- term care facilities starting in mid-December 2020 with a broader roll out of vaccines to health care workers and the general population starting in March/ April 2020.*

3.It might be beneficial to explain to non-canadian readers what access to care for mental health through the health system looks like and if there is any difference for a physician or not. In the U.S, the argument might be there is more access to care for physicians than non-physicians but as that access to care is through their employer, or employee access plans, perhaps many people go outside of the system to not be seen in relation to work and it would be hard to track. This is also especially important as later you say psychiatry might be used more by physicians due to access to specialty care.

**Author response:** Thank you for highlighting that an international audience will be less familiar with the Canadian healthcare system. Ontario has a universal single payer health system and all physician visits are covered. Importantly, access to psychiatry in Ontario is limited with long waitlists. We have moved a sentence from the methods on the Canadian health system to the introduction and expanded it to help the reader understand our data.

*“These databases capture essentially all outpatient visits to a physician and emergency department visits and hospitalizations through the provinces single payer universal health system”*

Methods:

4. Appreciated the large sample and controlling for specific variables. Would wonder if there was any lag in the increase over the pandemic time period for physicians seeking help and if a lot of it has come in more recent months. Would also wonder if there was any decrease around times of high covid spikes and whether that was even the opposite of the non-physicians (non physicians had more need, got more help, physicians had the need but had to help others). This might be outside the scope of the paper, but many people have argued that there was a delay due to physicians having to focus on taking care of others (even with increasing access).

**Author response:** This is an important consideration. Our study captured the first 18 months of the pandemic and occurred before key later developments such as the Omicron variant in December 2021. Importantly, our study frame did include two “interludes” between waves where physicians on the front line would have been able to seek care as patient loads decreased. We don’t see any clear visual pattern of differences in care seeking during these periods but agree that ongoing surveillance is indicated.

Context about the waves has been added to the methods and we have added to the discussion that ongoing surveillance in later stages of the pandemic are indicated.

*We suspect the physician mental health burden will continue after the COVID-19 pandemic as physicians work through critical backlogs (e.g., surgical and cancer care) with sicker patients and increase workloads and staffing shortages – as physicians reduce their practice hours or leave the profession.(31–33) Consequently, ongoing surveillance on physician mental health during later stages of the pandemic and recovery is indicated.*

Results/Discussion:

5.Why do the authors think that physicians sought care at higher numbers in comparison to non-physicians even pre-pandemic? Yes, this negligible with adjusted rates, but even being the same as the general population seems like it would be unexpected.

**Author response:** We outline in the discussion that physicians actually self -report higher levels of burnout than non-physicians.

*“Previous studies have found that physicians' self-reported mental health and well-being (e.g. burnout and work-life dissatisfaction) are worse compared to non-physicians[18,19]”*

6. Why do the authors think psychiatrists had higher rates of using psychiatrists? Is it simply that they are psychiatrists? Was there any difference pre/post pandemic in this group that would explain carrying the burden of this increase in people needing psychiatry over this time? This seems worth mentioning as to sustain care for the population, psychiatrists need to be mentally well. Even just mentioning that in the discussion seems important

**Author response:** There is data suggesting that the high numbers of visits by psychiatrists might be partly explained by studies showing that many psychiatrists report routinely attending therapy to improve their personal and professional growth. We have expanded on this in the discussion and at the editors discretion are happy to expand further at the editors discretion,

*“Interestingly, we found physicians had much higher psychiatry visits rates than non-physicians and lower primary care visit rates which persisted after excluding psychiatrists (whom we previously documented access psychiatry services at very high rates)[10]”*

Hadjipavlou  G, Halli  P, Hernandez  CAS, Ogrodniczuk  JS.  Personal therapy in psychiatry residency training: a national survey of Canadian psychiatry residents.   *Acad Psychiatry*. 2016;40(1):30-37. doi:[10.1007/s40596-015-0407-9](http://dx.doi.org/10.1007/s40596-015-0407-9)

7. p.18: Is it possible physicians lie about their substance use so it isn't coded as a problem? Especially because of licensing concerns? Is anxiety more acceptable of a diagnosis?

**Author response:** We think that this is definitely a possibility. We have included it as a possibility in our paper see page x lines y.

*Finally, we found a much higher proportion of outpatient MHA visits were related to drug and alcohol use in non-physicians (approximately 20% of visits) than physicians (approximately 5% of visits). This finding may reflect lower levels of substance use among physicians or a reluctance of physicians to seek help for addictions services due to*

*fear of licensing implications and stigma (27)*

8. p.19: Did Ontario/Canada do anything specific to change access to care for physicians over the pandemic that might make access explain the increase? Yes, telehealth helped, but that should've helped all groups. Some hospital systems increased their programs for mental health in healthcare workers over the pandemic and if this increased use/care it is worth mentioning as then this is worth the investment/sustainability (and something to say in discussion/conclusion).

**Author response:** Please see our response to the statistics editor comment 12 for full details. Changes in virtual care were universal and not specific for physicians. One way to contextualize the 4x greater relative increases in physician and non-physicians is that pre-pandemic physicians had half the rate of virtual care use which inflates the relative increase. This pre-pandemic discrepancy is likely because virtual care was delivered specifically for individuals in rural and remote settings and for those with substantial mobility concerns/ chronic illness.

We do agree that virtual care for physicians likely reduces multiple barriers to access including reducing stigma and being easier to schedule. We have added these comments to the discussion about the potential role of ongoing support for virtual care and possibly occupational physician support programs.

*“Our research suggests that an important intervention to help improve physician health and access to mental health services would be to continue virtual mental health services”*

9. p.19: In the conversation about reduction of barriers, what about actual anti-stigma, normalization campaigns by hospital systems and more awareness/acceptance of need in this group? Care seeking might increase if it is talked about more even through social media/the news and this is worth mentioning as again, then it could be worth investing in and sustaining. Did Ontario/Canada do anything specific in this realm worth mentioning?

**Author response:** Thank you, this is an important consideration. We have added the following to the discussion.

*“Finally, these findings may also be due to a reduction in mental health stigma among physicians due to the COVID-19 pandemic. During COVID-19, there was frequent messaging by governments and physician organizations concerning the mental health impacts of the pandemic. It’s possible that conversations regarding physician mental health during the pandemic reduced stigma and increased treatment access.”*

10. p.19- You discuss a systems investment as part of a solution, but what systems level investment is needed to sustain an increasing need/access to care and continue to decrease barriers to care? Given the focus of the paper, consider things like opt-out screening, COPE columbia program, as a guide. This seems more important to focus on as a conclusion/take home than the systems causes of mental health problems to the focus of your paper.

**Author response:** We agree that our conclusions should be more specific. We have added the following to our conclusion.

*A wide variety of interventions including increasing treatment access and system-level changes such as improving work-life balance and reducing the stress and administrative burden of physician work have been proposed and will require a substantial increase in health system investment.(3)*

Thank you for your work!

**Author response:** Thank you so much for your helpful comments and support for the paper.


Reviewer #3: Thank you for allowing me to review this revision. This study looked at psychiatry visits amongst physicians compared to non-physicians during the COVID-19 pandemic. Overall an important topic with significant implications for the healthcare workforce. Comments below:

1. unclear if Canadian system all care is accessed and readily available in this database searched. For example in the US many providers may pay cash to avoid the "stigma" of having a mental health visit notched in their record. Could this have been a possibility in this sample?

**Author response:** Thank you for highlighting that an international audience will be less familiar with the Canadian healthcare system. Essentially the answer to your question is no - physicians will *not* have seen other physicians and paid through means other than Ontario’s universal health coverage (OHIP). In Ontario, it is legal for a physician to charge patients directly for health services (e.g. someone could have paid cash for a visit). However, it is illegal for a physician to charge a higher fee than would be paid by OHIP. In addition, it is illegal for a physician who is seeing patients covered by OHIP (e.g. all Physicians in Ontario) to directly charge them for the services.

Importantly, our study does not capture visits to non-physician providers, such as psychologists, who are covered under private insurance and could also be paid for out of pocket. We do outline this in our limitations section - particularly since access to such services were increased during the pandemic.

2. With COVID-19 dates, were volumes similar to other countries during that time? I know 3/11 was the WHO declaration but how was the situation in Ontario? Has implications on the duration of the proposed "exposure" (e.g covid stress) that the providers were or not facing. May have varied geographically within the province as well.

**Author response:** We agree that additional information about how COVID-19 occurred in Ontario would be helpful. We have added this to the study design and setting in the methods.

*The first 18 months of the COVID-19 pandemic in Ontario were broadly characterized by three “waves” of infection and consequent burden on the health system (March 2020 to May 2020, mid-September 2020 to February 2021, and March 2021 to May 2021) with two periods of relatively lower levels of infections (June 2020 to September 2020, and June 2021 to August 2021). Vaccines in Ontario became available to health care workers in the highest risk settings and residents of long- term care facilities starting in mid-December 2020 with a broader roll out of vaccines to health care workers and the general population starting in March/ April 2020.*

3. why only family medicine and psych as the speciality? What about other specialities with whom covid care burden would have been high during the initial phase or surges of the pandemic  (e.g critical care, emergency medicine).

**Author response:** To clarify, we included outcomes for physicians regardless of specialty e.g. surgeons, internal medicine, pediatrics etc. Our primary outcome was a visit to a family physician for mental health reasons or a visit to psychiatrist for a mental health reason. The specialty breakdown at baseline is included in Table 1 and we have indicated in the methods more clearly that all specialties were included.

*“We also obtained physician specialty information for all physicians (Methods Supplement eTable2).”*

4. can you discuss a bit more about implications for this work and efforts aiming to destigmatize mental health amongst clinicians

**Author response:** Thank you for this suggestion. We have added the following section to the discussion/

*“Finally, these findings may also be due to a reduction in mental health stigma among physicians due to the COVID-19 pandemic. During COVID-19, there was frequent messaging by governments and physician organizations concerning the mental health impacts of the pandemic. It’s possible that conversations regarding physician mental health during the pandemic reduced stigma and increased treatment access.”*

Reviewer #4: Thank you for giving me the opportunity to review this manuscript. Mental health problem is an important problem among physicians during COVID-19 pandemic. The topic of this manuscript is interesting and have clinical implementation. However, there are some concern for this manuscript. 

1.      The first concern is that as a population-based cohort study, the author should give the flow chart to describe the maintenance information and lost to follow-up of the cohort etc.

**Author response:** Below is a flow chart showing how the cohort was created. At the editor’s discretion we are happy to include this in the supplement.

2.      The author did not present the detailed demographic information, did not distinguish the mental health history and new onset of mental health information. The mental health visit information mixed by many other confounders.

**Author response:** We present detailed information on the cohorts in Table 1 along with past mental health history. For example, in the 2 years prior to the onset of the COVID-19 pandemic 6.0% of physician had one or more outpatient appointments with a psychiatrist, 15.2% had one or more outpatient mental health visits with a family physicians, almost none (0.2%) had a visit to the ED or a hospitalization for a substance use or mental health condition. We agree that in this paper we did not distinguish whether increases in visits represented greater frequency of visits for individuals with a pre-pandemic mental health disorder (e.g. did mental health for people with prior conditions get worse during the pandemic) compared to new onset mental health visits. In our prior publication we did address this question finding that increases in outpatient mental health and addiction visits by physicians during COVID-19 were a mixtures of the two categories. <https://jamanetwork.com/journals/jamanetworkopen/fullarticle/2788289>

We have added this important clarification to the discussion

*“However, physicians had a larger increase in MHA visits than non-physicians and in prior work we showed that increases in MHA visits in physicians during the pandemic were driven both by greater incident mental health visits and greater intensity of mental health service use for those with pre-existing mental health conditions.”*

3.      The current mental health visit information analysis could be aided by the analysis of prescribing of psychiatric drugs. The authors are recommended to add the information of antidepressant, or antipsychiatry drugs pre pandemic and during COVID-19.

**Author response:** Thanks for the suggestion we agree that the prescribing of psychiatric drugs is important. ICES data included detailed health care data for all residents of Ontario but only included data on the prescription of controlled substances (e.g. opioids, benzodiazepines) or prescriptions for individuals with universal pharma coverage (e.g. aged 65+, or receiving financial aid from the government). We have a separate paper examining prescriptions of controlled substances in this cohort and unfortunately because most of our cohort is < 65 years of age, we have no reliable population-level data on prescriptions for drugs like antidepressants in this physician population.
