# Supplementary material for: Mental health and addiction health service use by physicians compared to non-physicians before and during the COVID-19 pandemic: A population-based cohort study in Ontario, Canada
Source: PLoS Med. 2023 Apr 18;20(4):e1004187. doi: 10.1371/journal.pmed.1004187 (PMC10112788; doi:10.1371/journal.pmed.1004187)
Supplement: Attachment — Submitted filename: Response January 25, 2023.docx [file pmed.1004187.s015.docx]

January 25, 2023

RE: PMEDICINE-D-22-02286RR: "Outpatient mental health and addiction visits by physicians compared to non-physicians before and during the COVID-19 pandemic.”

**Requests from Editors (dated January 25, 2023)**

1.Your Response to Reviewers should be uploaded as a single, individual file. Please remove your Response to Reviewers from your Cover Letter.

Author Response: Completed

2. Please upload a file showing your changes either highlighted or using a track changes function in your word processor. This document should be uploaded as the item type 'Revised Article with Changes Highlighted.' This will enable a swift editorial assessment of your manuscript. If you are submitting a PDF please ensure that the changes are clearly highlighted.

Author Response: Completed

3. We have amended your Competing Interest statement to comply with journal style. We kindly ask that you double check the statement and let us know if anything is incorrect.

Author Response: I approve of the amended Competing Interest Statement.

4. Please amend your detailed Financial Disclosure statement on the online submission form. This is published with the article. It must therefore be completed in full sentences and contain the exact wording you wish to be published.

- State the initials, alongside each funding source, of each author to receive each grant. For example: ""This work was supported by the National Institutes of Health (####### to AM; ###### to CJ) and the National Science Foundation (###### to AM).""

Author Response: Completed. Please note that the following two funding statements are mandated by our organization. “*Infrastructure to conduct the study was supported by the ICES (formerly known as the Institute of Clinical Evaluative Sciences), which is funded by an annual grant from the Ontario Ministry of Health and Ministry of Long-Term Care. Parts of this material are also based on data and information compiled and provided by the Canadian Institutes of Health Information*.”

- State what role the funders took in the study. If the funders had no role in your study, please state: “The funders had no role in study design, data collection and analysis, decision to publish, or preparation of the manuscript.”

Author Response: Completed.

- Please ensure that the funders and grant numbers match between the Financial Disclosure field and the Funding Information tab in your submission form. Note that the funders must be provided in the same order in both places as well.

Author Response: Completed.

- -The Acknowledgement section should not be a part of the Financial Disclosure on the submission form, only grant/funding received for this work.

Authors Response: Completed. Removed acknowledgements.

**Requests from Editors:**

1. Please include the additional sensitivity analyses conducted in response to Reviewer 1 in the Supporting Information and reference them in the Methods and Results.

Author Response: This has been completed

2. Related to the above, changes in the analysis-- including those made in response to peer review comments-- should be identified as such in the Methods section of the paper, with rationale.

Author Response: This has been completed

3. Please also include the beta coefficients (Reviewer 1 Comment 11) in the Supporting Information.

Author Response: This has been completed

4. Please also include the flow chard (Reviewer 4, Comment 1) in the Supporting Information.

Author Response: This has been completed

5. Please include the study setting (Ontario) in the title.

Author Response: This has been completed

Please remove the ‘Disclosures’ from the title page and the ‘Funding’ section from the end of the manuscript (these are captured as metadata via the Submission Form so please ensure all relevant information is captured on resubmission).

Author Response: This has been completed

In the last sentence of the Abstract Methods and Findings section, please describe the main limitation(s) of the study's methodology.

Author Response: This has been completed

At this stage, we ask that you include a short, non-technical Author Summary of your research to make findings accessible to a wide audience that includes both scientists and non-scientists. The Author Summary should immediately follow the Abstract in your revised manuscript. This text is subject to editorial change and should be distinct from the scientific abstract. Please see our author guidelines for more information: <https://journals.plos.org/plosmedicine/s/revising-your-manuscript#loc-author-summary>

Author Response: This has been completed

Please ensure all citations precede punctuation (e.g., line 152).

Author Response: This has been completed

Please cite all items in the Supporting Information according to our guidelines: <https://journals.plos.org/plosmedicine/s/supporting-information>

Author Response: This has been completed

Related to the above, please ensure all items in the Supporting Information are numbered correctly (e.g., possible issues at lines 214, 260, 311, and 344).

Author Response: This has been completed

Please include the numerator and denominator when reporting percentages (e.g., lines 266 and 269).

Author Response: This has been completed

Please ensure all abbreviations used in Tables/Figures are defined in the respective legends.

Author Response: We have uploaded revised figures with a label for MHA and larger text for the axes.

Please enlarge the text used in the key/axis labels in Figures 1 and 2.

Author Response: We have uploaded revised figures with a label for MHA and larger text for the axes.

Please provide the date of citation for internet sources (e.g., References 11 and 22).

Author Response: This has been completed

Comments from Reviewers:

Reviewer #1: Thanks for the revised manuscript and responses to my review. Apologies for not picking up that age was included as a continuous variable. Including the sensitivity analysis as a supplement would be fine with me as the results are very similar to including it in the linear form. I agree that use of the Poisson model to estimate RR here with individual level counts of health service utilisation should be fine and check of residuals is not needed. The revisions and responses to my queries have resolved my initial review. I recommend that the flowchart produced at R4's request be included as an appendix for the study.   

Author Response: Thank you for your review of this paper. We have included the study flow chart.

Reviewer #2: Thank you for your work to improve upon your manuscript based on the reviews. Really appreciate the contextualizing of the data in time and location.

My only question is that physicians seeking care at higher numbers pre-pandemic, doesn't align solely with there being greater need. There has always been high need in physicians, higher than other groups, but due to systemic and cultural factors, they don't seek care and studies have shown that they seek care in low numbers. Is there another explanation for this service usage? Or, can you say that it is not exclusively because of the higher need/rates and more studies are indicated to understand more?

Author Response: Thank you for the review of our paper and for the final comment. To clarify the crude rate of outpatient MHA visits in physicians was higher than non-physicians but this difference disappeared after adjustment for age, sex, neighbourhood income, and rural residency. We have clarified this in the manuscript

See page 20 lines 432-433.

“We found that pre-pandemic physicians had similar rates of MHA outpatient visits relative to non-physicians after accounting for differences in sociodemographic characteristics.”

Reviewer #3: The authors have addressed my comments and I have no additional comments. Thank you very much to the author team!

Author Response: Thank you very much for your review of this paper.
